# Bacteriology as a Fad

**Published:** 1899-11

**Authors:** Emmet L. Smith

**Affiliations:** Chicago


### BACTERIOLOGY AS A FAD.

CHICAGO, Nov. 26th, 1899.

Editor THE HOMŒOPATHIC PHYSICIAN, Philadelphia, Pa.

Dear Sir.—I notice in your October number at page 474, that you publish the article from *Home Magazine* and *Medical Times* about "Bacteriology as a Fad."

I take pleasure in calling your attention to this (Nov.) issue of *Medical Times*, where Dr. Gibbs *denies* the article. Even with this denial, there will be a certain class of journals, publish it nevertheless, and can expect to see this as well as other quotations similarly denied, used as *stock quotations* from now on by those who do not know the difference between harmful and helpful bacteria.

Respectfully,

EMMET L. SMITH.

